# Aquatic nitrous oxide reductase gene (*nosZ*) phylogeny and environmental distribution

**DOI:** 10.3389/fmicb.2024.1407573

**Published:** 2024-05-21

**Authors:** Naomi Intrator, Amal Jayakumar, Bess B. Ward

**Affiliations:** Department of Geosciences, Princeton University, Princeton, NJ, United States

**Keywords:** nitrous oxide, *nosZ*, oxygen minimum zone, nitrogen cycling, denitrification

## Abstract

Nitrous oxide (N_2_O) is a potent greenhouse gas and a major cause of ozone depletion. One-third of atmospheric N_2_O originates in aquatic environments. Reduction of N_2_O to dinitrogen gas (N_2_) requires the nitrous oxide reductase enzyme, which is encoded by the gene *nosZ*. Organisms that contain *nosZ* are the only known biological sinks of N_2_O and are found in diverse genera and a wide range of environments. The two clades of *nosZ* (Clade I and II) contain great diversity, making it challenging to study the population structure and distribution of *nosZ* containing organisms in the environment. A database of over 11,000 *nosZ* sequences was compiled from NCBI (representing diverse aquatic environments) and unpublished sequences and metagenomes (primarily from oxygen minimum zones, OMZs, where N_2_O levels are often elevated). Sequences were clustered into archetypes based on DNA and amino acid sequence identity and their clade, phylogeny, and environmental source were determined. Further analysis of the source and environmental distribution of the sequences showed strong habitat separation between clades and phylogeny. Although there are more Clade I *nosZ* genes in the compilation, Clade II is more diverse phylogenetically and has a wider distribution across environmental sources. On the other hand, Clade I *nosZ* genes are predominately found within marine sediment and are primarily from the phylum Pseudonomonadota. The majority of the sequences analyzed from marine OMZs represented distinct phylotypes between different OMZs showing that the *nosZ* gene displays regional and environmental separation. This study expands the known diversity of *nosZ* genes and provides a clearer picture of how the clades and phylogeny of *nosZ* organisms are distributed across diverse environments.

## 1 Introduction

Nitrous oxide (N_2_O) is a potent greenhouse gas, with a ~300-fold greater global warming potential per molecule compared to carbon dioxide on the 100 year timescale, and a significant player in ozone depletion (Ravishankara et al., 2009; Felgate et al., 2012). Biological reduction of N_2_O to dinitrogen gas (N_2_) requires the enzyme nitrous oxide reductase (N_2_OR), which is encoded by the gene *nosZ* (Zumft, 1997). Organisms that contain *nosZ* are the only known biological sinks of N_2_O. The ability to reduce N_2_O has been found in a wide range of genera and environments (Jones et al., 2008). The widespread distribution and high abundance of *nosZ* containing organisms across environments indicates the ability to reduce N_2_O is an ecologically important trait. The prevalence of *nosZ* implies a potential N_2_O sink, thereby impacting net N_2_O emissions (Jones et al., 2012).

One-third of atmospheric N_2_O originates in aquatic environments, predominantly from microbial metabolism (Ciais et al., 2013), and ~20% of global N_2_O emissions are estimated to come from natural processes in the global ocean (Gluschankoff et al., 2023). N_2_O can be formed both biotically through processes such as denitrification, nitrification, codenitrification, and nitrfier-denitrfication as well as abiotically through chemodenitrification. In the ocean, oxygen minimum zones (OMZs), regions where dissolved oxygen concentrations reach low ( ≤ 20 μM; Lam and Kuypers, 2011) or zero concentrations, are significant sources of N_2_O (Nevison et al., 2004; Arévalo-Martínez et al., 2015; Yang et al., 2020; Gluschankoff et al., 2023). *NosZ* is often associated with canonical denitrification, also referred to as complete denitrification. The biological reduction of N_2_O is the final step in the denitrification pathway in which fixed nitrogen is reduced through anaerobic respiration in which nitrate (${\text{NO}}_{3}^{-}$), nitrite (${\text{NO}}_{2}^{-}$), nitric oxide (NO), and N_2_O are successively reduced to dinitrogen gas (N_2_) (Zumft, 1997; Felgate et al., 2012). However, the capacity and genetic potential for N_2_O reduction through N_2_OR likely extends beyond known denitrifying organisms such that the scope of natural N_2_O-reducing microbial community is likely greatly underestimated (Chee-Sanford et al., 2020). Denitrification is assumed to regulate oceanic N_2_O concentrations because this pathway is both a sink and a source of nitrous oxide (Granger and Ward, 2003). This study aims to characterize *nosZ* in marine and other aquatic microbial communities that can remove N_2_O from the environment.

Although *nosZ* has been studied in cultivated organisms for decades (Zumft, 1997), much is still unknown about the distribution, taxonomy, function, and biogeochemical impacts of *nosZ*-containing organisms within the environment (Sanford et al., 2012; Hallin et al., 2018; Bertagnolli et al., 2020). This knowledge gap is partly due to the high taxonomic diversity of *nosZ*, which has been revealed in the environment through multiple approaches including PCR amplification and sequence analyses (Jones et al., 2012; Sanford et al., 2012; Orellana et al., 2014; Hallin et al., 2018; Chee-Sanford et al., 2020; Zhang B. et al., 2021; Wei et al., 2023). New studies are continuously adding to the *nosZ* diversity implying that novel sequences are likely still undiscovered.

*NosZ* has a complex evolutionary history. Studies comparing the phylogeny of denitrification genes found them to be incongruent with 16S rRNA suggesting horizontal gene transfer (HGT) as the potential cause behind this disagreement (Delorme et al., 2003; Dandie et al., 2007; Jones et al., 2008). However, compared to other denitrification genes, *nosZ* has been found to have the highest level of congruence with 16S rRNA taxonomic classifications (Dandie et al., 2007; Zumft and Körner, 2007). Therefore, other mechanisms such as gene duplication and divergence or lineage sorting may better explain *nosZ*'s evolutionary history. If HGT occurred during the evolution of *nosZ*, it likely transpired between closely related organisms (Jones et al., 2008).

Two phylogenetically distinct variants of *nosZ* have been identified: Clade I and II, also known as typical and atypical, respectively (Jones et al., 2012; Sanford et al., 2012). Differences in kinetics (Yoon et al., 2016; Tang et al., 2022), community composition, and/or environmental distribution between Clade I and II containing organisms are likely to impact environmental N_2_O fluxes. Clade I organisms are often found to also contain genes encoding the enzymes for complete denitrification. In contrast, Clade II organisms typically lack one or more of the other denitrification enzymes and are therefore often referred to as “incomplete denitrifiers” (Sanford et al., 2012; Graf et al., 2014). Additional research is necessary to better understand the biogeochemical impacts of the two clades on global microbial N_2_O fluxes.

The majority of previous research examining *nosZ* focuses on terrestrial sources, predominately soils, leaving *nosZ* understudied in marine and other aquatic environments including hydrothermal vent systems, salt marshes, wastewaters, freshwater and estuary systems. We surveyed published databases and metagenomic datasets of three major marine OMZs to focus on *nosZ* sequences from marine and other aquatic environments. *NosZ* containing organisms have the potential to regulate N_2_O emissions that contribute to ozone depletion and the greenhouse effect; therefore, characterization of their diversity and environmental distribution will help to understand their biogeochemical impacts. This study analyzes the diversity and distribution of *nosZ* focusing on the environmental sources of the gene to provide further insight into what environments and taxa are involved in the reduction of N_2_O.

## 2 Materials and methods

### 2.1 Database compilation

To encompass the full range of diversity of the *nosZ* gene, all gene features and CDS (coding sequences) of annotated *nosZ* nucleotide sequences between 500–3,500 bp were downloaded from the National Center for Biotechnology Information (NCBI). Genbank database on June 1^st^, 2021 (28,866 sequences). These *nosZ* gene sequences were extracted from whole genomes as well as fragments from environmental clones and metagenomes. To extract these sequences Hidden Markov Models (HMM) for *nosZ* sequences were generated using a custom curated subset of *nosZ* sequences from NCBI and used to search assembled reads of metagenomes from the Arabian Sea (AS, during late Monsoon/intermonsoon transition in September–October 2007) and Eastern Tropical North (ETNP in 2016 and 2018) and South Pacific (ETSP in 2013) using HMMR (v3.3.2) (Eddy, 1998) (Supplementary Table 1, Supplementary Figure 1). HMMR recovered 255 new sequences from these sources with the inclusion threshold of an E value of 0.01 or less and a per-domain conditional E-value of 0.01 or less.

Additional *nosZ* sequences from the Chesapeake Bay in 2020 and ETNP in 2018 were obtained from clone libraries of PCR products amplified using clade specific primers. PCR products (~699 bp) for Clade I were amplified using NosZ1F (5′-WCS YTG TTC MTC GAC AGC CAG-3′) and NosZR (5′-CAT GTG CAG NGC RTG GCA GAA-3′) (Henry et al., 2006) and for Clade II, a ~745 bp product using nosZ-II-F: (5′-CTI GGI CCI YTK CAY AC-3′) and nosZ-II-R36 (5′-GCI GAR CAR AAI TCB GTR C-3′) (Jones et al., 2012). This resulted in 107 and 268 novel *nosZ* sequences found from the Chesapeake Bay in 2020 and ETNP in 2018, respectively. Lastly, sequences from Jayakumar et al. (2018) and Bertagnolli et al. (2020) were added to the compilation. Duplicate sequences were then removed from the compilation of all the sequences listed above using SeqKit (v.2.5.1; Shen et al., 2016) by keeping only the first instance of each sequence and its accession number, resulting in a total of 11,095 *nosZ* sequences.

### 2.2 Database processing

The compilation of *nosZ* sequences was clustered into operational taxonomic units (OTUs) using CD-HIT (Li and Godzik, 2006; Fu et al., 2012) with a defined similarity threshold of 87% sequence identity (Taroncher-Oldenburg et al., 2003; Ward et al., 2007; Bulow et al., 2008), which generated 2,282 OTUs. This threshold was chosen in order to cluster homologous genes at the level found to differentiate functional genes at the approximate level of species distinction (Purkhold et al., 2000; Taroncher-Oldenburg et al., 2003). Six hundred seventy-four OTUs contained >2 sequences. The remaining OTUs containing one or two sequences were treated as individual sequences, which are identified by their accession numbers.

The environmental source of the sequences clustered within each OTU were determined by searching NCBI and published literature. Sequences were labeled with the following environmental sources: marine sediment, marine water column, marine OMZs, hydrothermal vent systems, terrestrial, animal (i.e., from the animal microbiome), salt marsh, freshwater and estuary systems, wastewater systems, and aquatic other. Other aquatic environments mainly included hypersaline and/or soda lakes and contaminated waters. These environments were classified as other because of the great variability between them. There were not enough sequences from each kind of these other environments to make clear conclusions. OTUs that did not contain any marine or aquatic sequences or sequences from Jayakumar et al. (2018) and Bertagnolli et al. (2020) were removed from further study. The remaining OTUs containing more than three sequences were aligned with MAFFT v7.407 (Katoh and Standley, 2013), and a consensus sequence for each OTU was generated with emboss v6.6.0 (Stamatakis, 2014).

An alignment including all of the consensus sequences and the individual sequences could not be made using the nucleotide sequences. Therefore, DNA sequences were converted to protein sequences using gene prediction program GeneMarkS (v.4.28; Besemer, 2001). Protein sequences were then aligned using Clustal Omega (v.1.2.4; Sievers et al., 2011). Because most of the sequences are *nosZ* fragments (usually cloned PCR products), they fell on either the 5′ or 3′ end of a full *nosZ* gene with only a small (~42 bp) region of overlap. Consequently, sequences were further separated into 5′ and 3′ groups for further analysis (Supplementary Figure 2). In the absence of complete continuous sequences the phylogenetic coherence between the 5′ and 3′ regions cannot be ascertained; therefore, the two regions were analyzed separately. Twenty-four clusters and 127 individual sequences, which were mainly extracted from whole genomes, spanned both the 5′ and 3′ regions and were included in both groups. The longest possible aligned 5′ and 3′ region within each alignment was extracted and used for further analyses.

The extracted aligned protein regions were converted back to nucleotide sequences and analyzed with the probe finding algorithm to form archetypes (Bulow et al., 2008). These aligned nucleotide sequences for each region were analyzed using a pairwise distance matrix. The 70 bp region which had the greatest average distance between sequences was selected for further analyses. Therefore, this algorithm determines the most distinct 70 bp region within the aligned region and was used to group the sequences into archetypes, each of which is represented by a unique 70-mer region. The 70-mer archetype region with an identity threshold of 87% provides the optimal discrimination between related sequences at approximately the species level for previously studied functional genes (Ward et al., 2007; Bulow et al., 2008). A 70-mer region is long enough to represent and distinguish amongst related types, but also allowed us to incorporate shorter sequences into the analyses, therefore, allowing us to obtain a high resolution of divergence and diversity. Compared to the reference sequence of the *Pseudomonas fluorescens*'s *nosZ* gene consisting of 1,920 bp (Philippot et al., 2001; AF197468.1), the 5′ 70-mer region is located around ~792–862 bp, and the 3′ 70-mer region is ~1,322–1,392 bp (Supplementary Figure 2).

The archetypes were identified as Clade I, II, or unknown based on phylogenetic inference by using NCBI's blastn and blastp (Altschul et al., 1990). Additional metadata were collected by searching with the archetype using blastn optimized for highly similar sequences on NCBI (Camacho et al., 2009). Archetype sequence information and metadata are listed in Supplementary Tables 2, 3. Phylogenetic trees were generated with the aligned 5′ and 3′ 70-mer archetype regions using RAxML v8.2.12 (Stamatakis, 2014) and viewed and edited in iTOL v6 (Letunic and Bork, 2006).

## 3 Results

### 3.1 Aquatic *nosZ* sequence compilation overview

A total of 11,095 *nosZ* sequences were collected as described above from the databases and from other published and newly obtained clone library analysis and metagenome searches. In addition, we searched assembled reads of metagenomes from the Arabian Sea (in 2007), ETSP (in 2013), and ETNP (in 2016 and 2018), and found 146, 16, 52 and 41 new *nosZ* sequences respectively. Furthermore, small clone libraries from the Chesapeake Bay and ETNP (2018) added 107 and 268 *nosZ* sequences respectively (Supplementary Table 1). Database compilation resulted in 3,942 total unique marine or other aquatic *nosZ* sequences that were further used for analyses. Most of the sequences were derived from PCR amplification but several new sequences were discovered in metagenomes.

**Figure 1:**
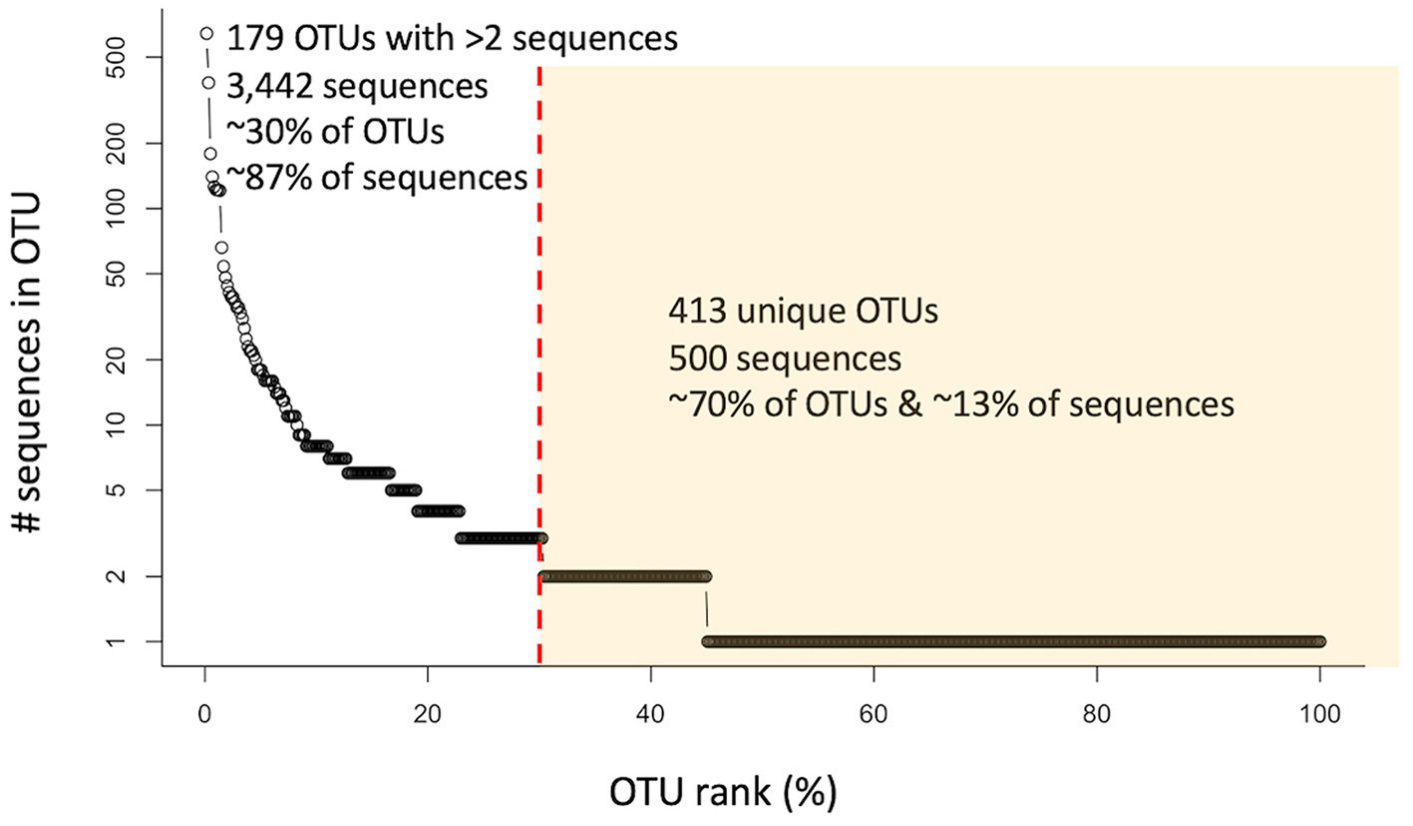
Rank abundance curve of 592 analyzed OTUs (circles) containing *nosZ* sequences from marine or other aquatic environments. Red dashed line separates OTUs containing more than 2 sequences from those containing 2 or less (unique/individual sequences) highlighted in yellow.

All the *nosZ* sequences (i.e., prior to separation into 5′ and 3′ groups) were clustered into 2,282 OTUs using CD-HIT (Supplementary Figure 3). However, only 592 clusters contained sequences from marine or other aquatic environmental sources and were included in further analyses. Of those, 179 OTUs contained more than 2 sequences. These 179 OTUs represent only ~30% of the clusters, but account for ~87% of the sequences (3,442 sequences). The largest cluster contains 643 sequences (~16% of the sequences). The remaining OTUs, the unique individual sequences, represent ~70% of the clusters and ~13% of the sequences (500 sequences). This distribution of sequences shows that while the majority of the clusters are unique, most of the sequences group into larger OTUs (Figure 1).

### 3.2 5′ region of *nosZ* gene

**Figure 2:**
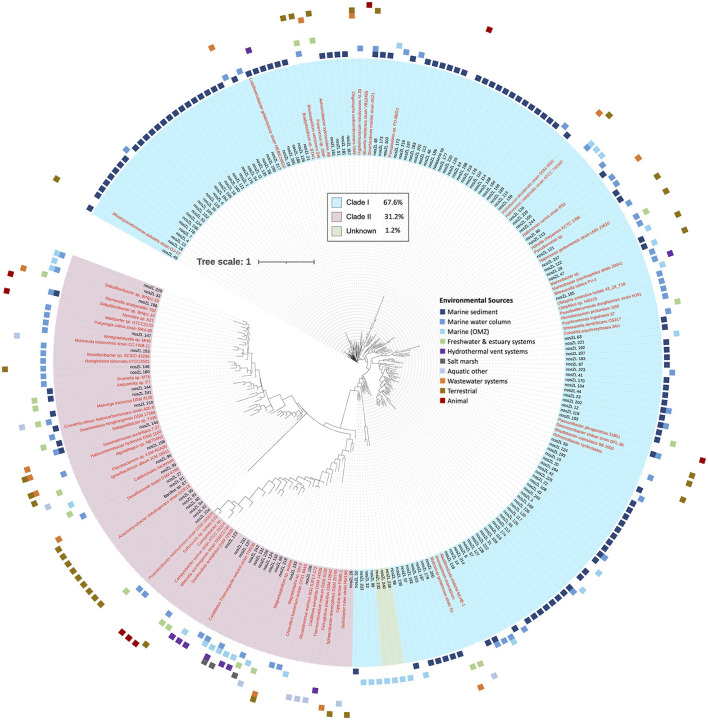
Phylogenetic tree of *nosZ* based on representative archetype nucleotide sequence from the 5′ region. Tree is based on 866 *nosZ* sequences. Names of nodes written in red represent cultured/known organisms. Colored squares on the outermost rim represents the environmental sources of the sequences within each archetype. The inner colored rim represents the achetype's *nosZ* clade assignment (key in black box).

A total of 866 sequences aligned at the 5′ region of the *nosZ* gene and were grouped into 250 archetypes, which were further examined. One hundred eighty-one archetypes represented 252 unique individual sequences only and therefore, did not contain clusters. However, the remaining 69 archetypes contained the majority of the sequences (614 sequences). The largest archetype represented 75 sequences. Archetypes were classified into Clade I (169 archetypes, 67.6%) and Clade II (78, 31.2%). Clade I archetypes represent 681 sequences, while Clade II represent 182 sequences. The clade affiliation of three archetypes could not be determined so they were classified as unknown. The unknown sequences were all single sequences obtained from marine OMZs. The clades therefore show very clear phylogenetic separation (Figure 2).

**Figure 3:**
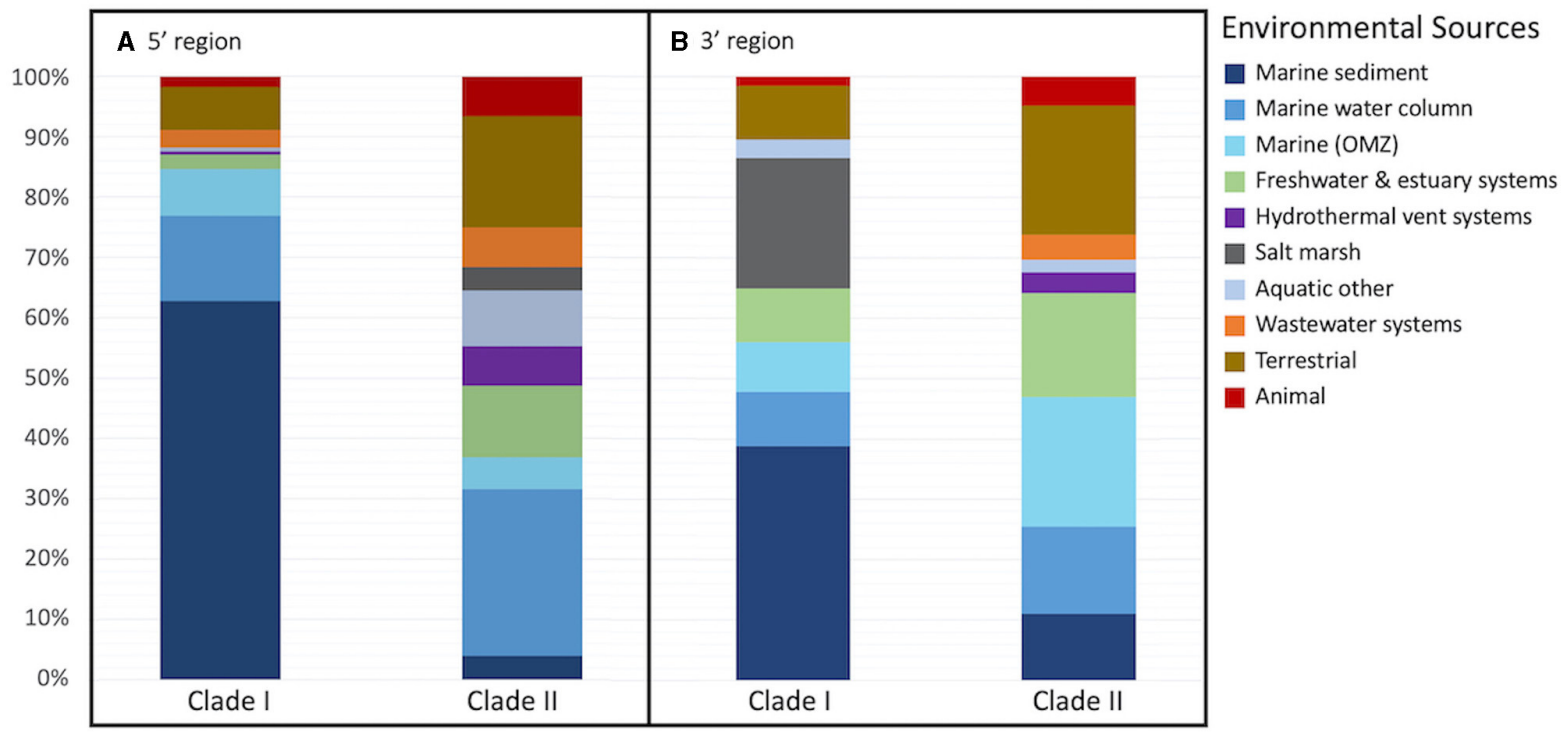
Percent of representative archetype sequences found from each environmental source. Bar plots display the percent of representative archetype sequences found from each environmental source for each *nosZ* clade **(A)** the 5′ region containing 250 archetypes and **(B)** the 3′ region containing 280 archetypes. For 3 of the 5′ region archetypes (obtained from marine OMZs) and 1 3′ region archetype (obtained from the marine water column) the clade could not be determined; they are not included in the figure.

The majority of archetypes contain sequences obtained from only one of the environmental sources (205 archetypes, 82%); no archetype contains sequences representing more than three different sources (36 represent two environmental sources and 8 represent three different environmental sources). Figure 2 depicts the phylogeny of the *nosZ* sequences of the 5′ region of the gene and shows the spread of environmental sources across archetypes and clades. The environmental sources for each archetype (based on the 70-mer archetype analysis) were identified, separated by clade, and the total number of archetypes representing an environmental source within each clade was determined (Figure 3A). Archetypes within Clade I were predominately obtained from marine sediment (62.7%) while archetypes of Clade II sequences derive from a much more diverse range of environmental sources, although the marine water column represents the largest representative environmental source (27.6%). Environmental sources of terrestrial, animal, wastewater systems, marine water column, hydrothermal vent systems, freshwater & estuary systems are found more often in Clade II *nosZ* genes.

Archetypes containing sequences retrieved from marine OMZs were examined further to resolve if there was smaller scale regional biogeography by comparing their occurrence within and among the three major OMZs (Table 1). The majority (76%) of archetypes containing OMZ sequences contained sequences from only one of the OMZ regions (i.e., either AS, ETNP, or ETSP). Twenty-four percent of the archetypes contained sequences from both the AS and ETNP, and no archetypes contained sequences from all three major OMZs. Therefore, most of the OMZ sequences appear to be unique to a specific region.

**Table 1:** Regional biogeography of marine OMZ archetypes.

**AS**	**ETNP**	**ETSP**	**AS and ETNP**	**AS and ETSP**	**ETNP and ETSP**	**AS and ETNP and ETSP**	**Total**
**(A) 5**′**region;** ***n*** = **25**							
28%	4%	44%	24%	0%	0%	0%	100%
76%			24%			0%	100%
**(B) 3**′**region;** ***n*** = **49**							
26.5%	24.5%	8.2%	34.7%	0%	2%	4.1%	100%
59.2%			36.7%			4.1%	100%

Table displays the percentage of archetypes with *nosZ* sequences obtained from each of the major marine OMZ or OMZs including **(A)** 25 archetypes of the 5′ region and **(B)** 49 archetypes of the 3′ region. Color depicts percentage of archetypes from high (red) to low (blue).

**Figure 4:**
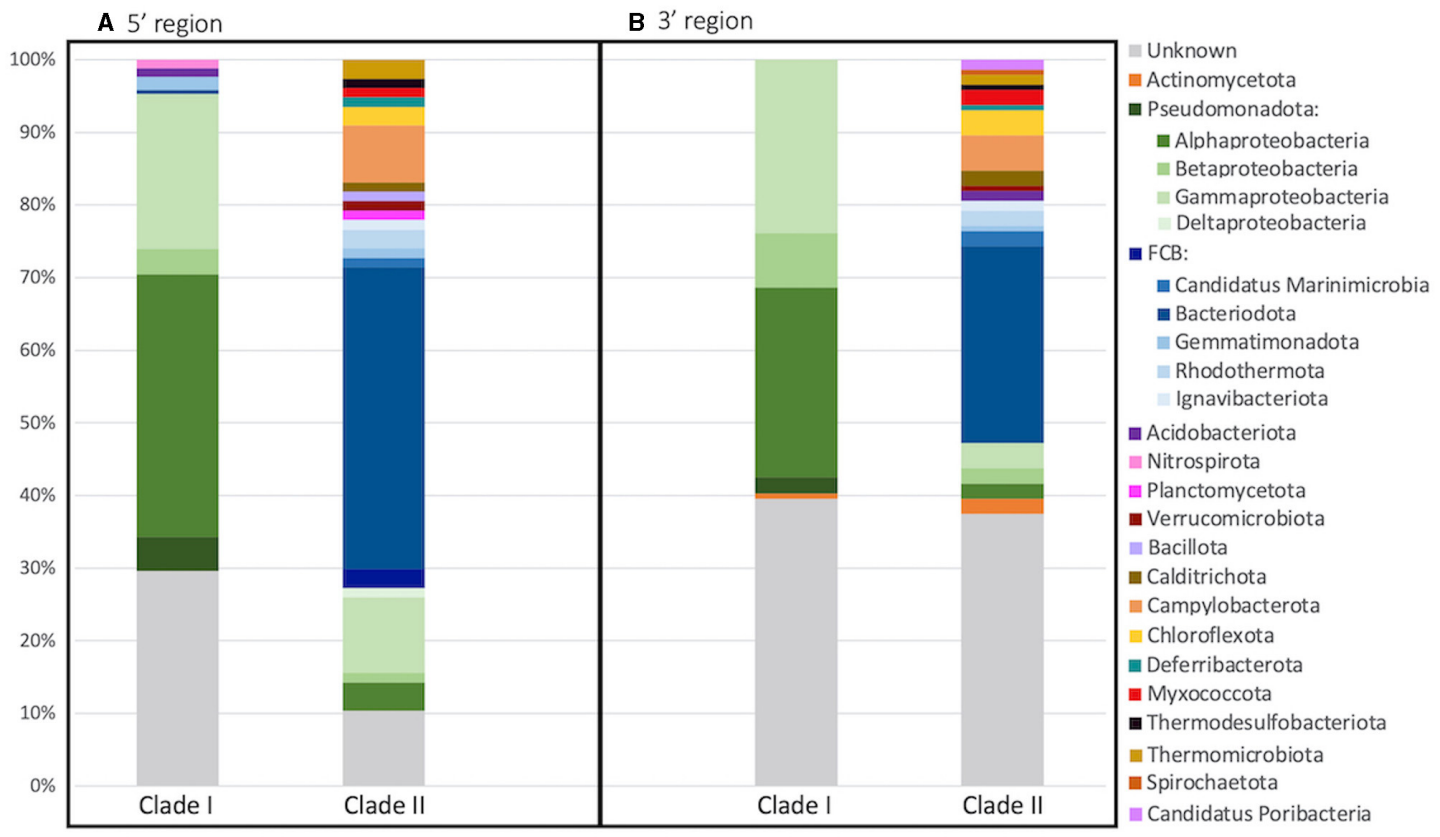
Archetype taxonomy between clades. Taxonomy of each archetype's representative sequence was determined through phylogenetic inference for the **(A)** 5′ and **(B)** 3′ region of the *nosZ* gene. Bar plot depicts the percentage of archetypes within each phyla for each clade. Superphylum FCB is divided into phylum in shades of blue and phylum Pseudomonadota is divided further into classes in shades of green.

The taxonomy of each archetype's representative sequence was determined through phylogenetic inference (Figure 4A). The phylum/class of both Clade I and II could be determined for a majority of the sequences, 70.4%/66.9% and 89.6%/83.1% respectively. However, only 20.7% of Clade I sequences could be assigned to the genus/family level and 64.9% for Clade II. Clade I primarily consisted of sequences from the Pseudomonadota phylum (65.7%), with most classified as Alphaproteobacteria (36.1%). On the other hand, Clade II was dominated by Bacteroidota sequences (41.6%). In addition, Clade II was more diverse, containing sequences from 16 different phyla, while Clade I only had five phyla, three of which were found in both Clades.

**Figure 5:**
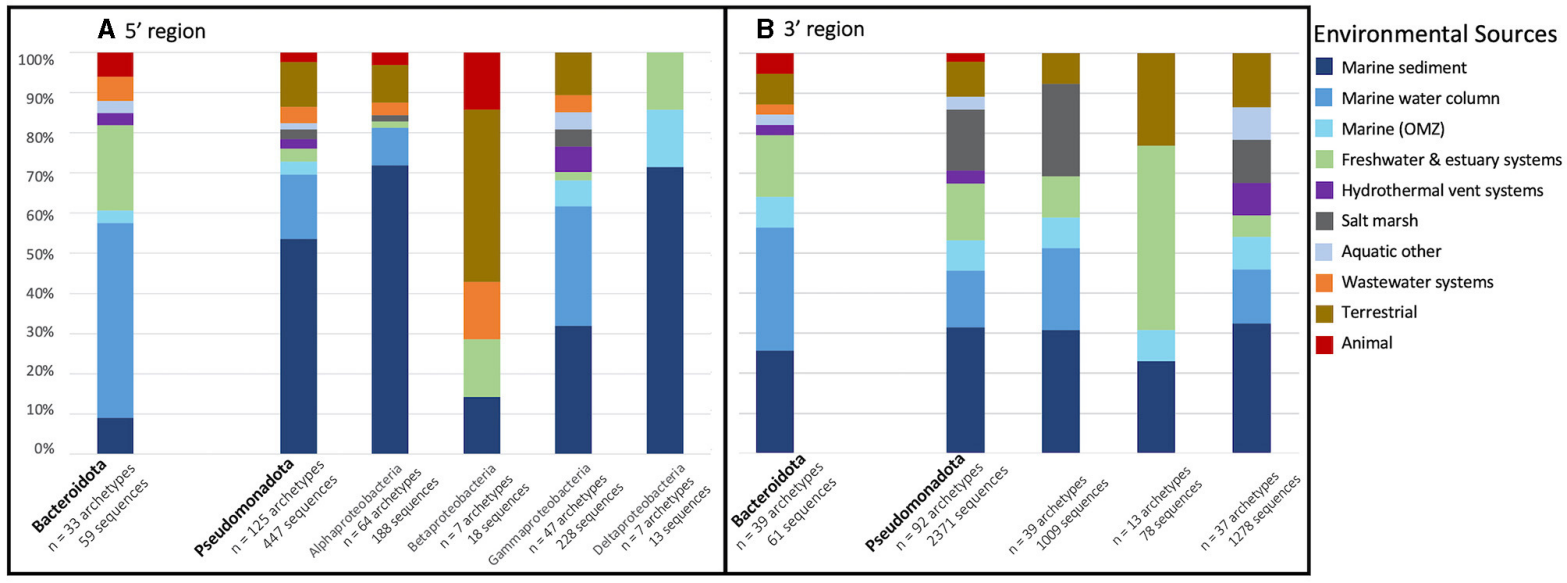
Percent of archetype environmental source of the two dominant phyla. Bar plot displays the percent of representative archetype sequences found from each environmental source of the two dominant phyla, Bacteroidota **(left)** and Pseudomonadota **(right)**, for **(A)** the 5′ and **(B)** 3′ region of the *nosZ* gene. Pseudomonadota is further divided into classes.

The two dominant phyla, Bacteroidata and Pseudomonadota, were examined further by determining what percent of archetypes came from each environmental source within each phylum (Figure 5A). Bacteroidota, which was represented in of 33 archetypes (59 sequences), was primarily obtained from the marine water column (~48.5%), followed by freshwater and estuarine systems (21.2%). On the other hand, Pseudomonoadota (125 archetypes representing 447 sequences) was mostly retrieved from the marine sediment (53.6%). The Pseudomonoadota classes were further analyzed. Alpha-, Gamma-, and Deltaproteobacteria were all predominately obtained from marine sediment (71.9%, 31.9%, and 71.4% respectively). Betaproteobacteria's main environmental source was terrestrial; however, it only comprises 7 archetypes and 18 sequences.

### 3.3 3′ region of *nosZ* gene

**Figure 6:**
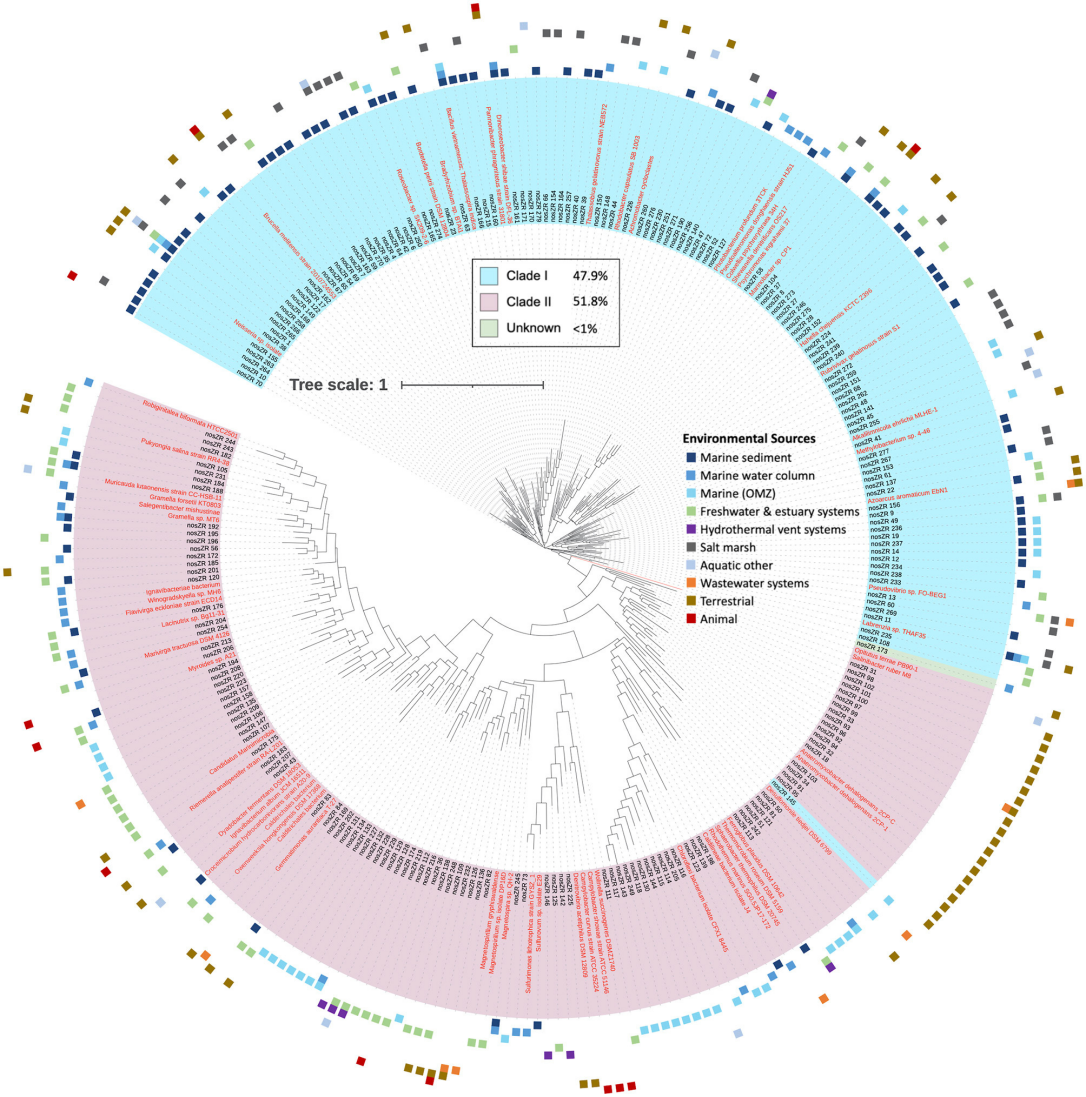
Phylogenetic tree of *nosZ* based on representative archetype nucleotide sequence from the 3′ region. Tree is based on 3,190 *nosZ* sequences. Names of nodes written in red represent cultured/known organisms. Colored squares on the outermost rim represents the environmental sources of the sequences within each archetype. The inner colored rim represents the achetype's *nosZ* clade assignment (key in black box).

The majority of the *nosZ* sequences aligned on the 3′ region of the gene. A total of 3,190 *nosZ* sequences grouped into 280 archetypes, which were further analyzed. Two hundred and twenty-four individual sequences made up 189 archetypes while most of the sequences (2,966 sequences) were clustered into 171 archetypes. The largest archetype represented 849 sequences. Archetypes divided almost equally between Clade I and II, 47.9% (134) and 51.8% (145) respectively, with only one archetype in which the clade affiliation could not be determined. Despite a nearly equal divide between Clade I and II archetypes, there are significantly more sequences represented within the Clade I archetypes vs. Clade II, 2,758 and 431 sequences respectively. Similar to the 5′ region of the *nosZ* gene, the clades separate out phylogenetically (Figure 6).

The environmental source for each archetype was determined. Like the 5′ region of the *nosZ* gene, most of the archetypes from the 3′ region (210 archetypes, 75%) represent only one environmental source. However, there are two archetypes represented by sequences from five different environmental sources (*Labrenzia sp*. and uncultured gammaproteobacterial), and one archetype representing six different environmental sources (uncultured alphaproteobacterial). The remainder of the archetypes represent three or fewer different environmental sources. The phylogenetic tree of the representative *nosZ* archetype sequences in the 3′ region of the gene in Figure 6 portrays the spread of the environmental sources across the clades. Figure 3B depicts spread of environmental sources for each archetype's representative sequence. Similar to the 5′ region of the *nosZ* gene, archetypes within Clade I were predominately obtained from marine sediment (38.8%), followed by salt marshes (21.6%). On the other hand, Clade II archetypes have a wider distribution across environmental sources. Among the Clade II archetypes the terrestrial and marine OMZ environmental sources were equally represented (21.38%) followed by freshwater and estuarine systems (17.2%). Environmental sources of terrestrial, marine OMZs, animal, wastewater systems, marine water column, hydrothermal vent systems, wastewater systems, and freshwater & estuary systems were found more often in Clade II *nosZ* genes.

Biogeography of the *nosZ* sequence was examined at a smaller scale by focusing on the archetypes that contained sequences retrieved from marine OMZs. Within each of these archetypes, the source of the sequences was determined, i.e., which of the major OMZs (AS, ETNP, or ETSP) (Table 1). Similar to the 5′ region, archetypes containing OMZ sequences from the 3′ region were predominately obtained (59.2%) from only one of the major OMZs. Fewer archetypes were retrieved from two distinct OMZs (36.7%). Only 4.1% archetypes contained sequences from all three of the major OMZs. This suggests the majority of *nosZ* sequences are unique to their environment.

Like the 5′ region of the *nosZ* gene, the majority of the sequences could be classified into phylum/class for Clade I (60.4%/58.2%) and Clade II (63.2%/54.2%) (Figure 4B). Additionally, only 23.1% of Clade I and 32.6% of Clade II sequences could be assigned to the genus/family. Like the 5′ region, most of the Clade I sequences are within the Pseudomonadota phylum (59.7%) with Alpha- and Gamma-proteobacteria dominating (26.1% and 23.9%). Clade II was again predominately Bacteroidota sequences (27.1%). Clade II had greater diversity consisting of sequences from 18 different phyla compared to sequences from Clade I which were found within only 2 different phyla.

As noted above, Bacteroidota and Pseudomonadota were the largest phyla containing *nosZ* sequences, as was also observed from the 5′ region. Within each phylum, the percent of archetypes derived from each environmental source was determined (Figure 5B). Similar to the 5′ region, Bacteroidota's (39 archetypes representing 61 sequences) main environmental source was the marine water column (30.8%); however, this is followed by marine sediment (25.6%) instead of freshwater and estuarine systems. As with the 5′ region, archetypes within the phylum Pseudomonadota (92 archetypes representing 2,371 sequences) were mostly obtained from the marine sediment (30.8%). Alpha- and Gammaproteobacteria were both predominately retrieved from marine sediment (30.7% and 32.4% respectively). Betaproteobacteria was mainly obtained from freshwater and estuarine systems; however, it was the smallest Pseudomonadota class in terms of both number of archetypes and sequences (13 and 78 respectively).

## 4 Discussion

### 4.1 *nosZ* diversity

Although the *nosZ* gene has been identified and studied for decades, knowledge of the function, distribution, and taxonomy of *nosZ* containing organisms in the environment is still fragmented (Hallin et al., 2018; Bertagnolli et al., 2020). This is in part due to the high taxonomic diversity of *nosZ*-containing organisms revealed through phylogenetic- and high throughput sequencing analysis of *nosZ* genes in natural communities (Jones et al., 2012; Sanford et al., 2012; Orellana et al., 2014; Hallin et al., 2018). The *nosZ* sequences analyzed in this study were so distinct at the nucleotide level, they could not be well aligned, further highlighting the *nosZ* diversity. However, the sequences were similar enough at the protein level such that they could be aligned, suggesting despite the great DNA level diversity, the genes encode fundamentally the same enzyme. The high level of similarity at the protein level implies strong functional constraints on the enzymes, which probably extends to ecologically significant features such as oxygen sensitivity and the requirement for copper at the active sites. While the gene sequence has evolved consistently with the host genome, the functionality of the protein has been conserved.

This great diversity of *nosZ* at the DNA level introduced detection and analytical challenges, which suggests that the extent of the N2O-reducing microbial community is still underestimated (Chee-Sanford et al., 2020). Over 600 new and widely divergent *nosZ* sequences were found in our searches of marine metagenomes and even in small clone libraries from estuarine and oceanic environments (Supplementary Table 1). It is clear that new studies are continuously adding to the *nosZ* diversity, and likely much diversity is still undiscovered. This great diversity is evident in Figure 1, while the majority of sequences group together into larger clusters, most of the clusters, ~70%, are unique. Even after further grouping into archetypes, a similar pattern is evident: the majority of the archetypes represent individual sequences for both the 5′ and 3′ region of the *nosZ* gene (72% and 68% respectively), meaning most of the branches on the phylogenetic trees (Figures 2, 6) are unique. While this study uncovered an extensive diversity of *nosZ* sequences, this rate of discovery implies that additional diversity not covered in this study is yet to be revealed.

### 4.2 Variation between Clade I and Clade II

#### 4.2.1 Clade abundance

Jones et al. (2012) identified two distinct groups of the N_2_OR protein, Clade I and Clade II. It has since been suggested that differences between Clade I and II organisms could impact N_2_O emissions. A kinetics study showed Clade I organisms to have higher half-saturation constants for N_2_O than Clade II organisms, suggesting Clade II would perform better at lower N_2_O concentrations (Yoon et al., 2016). Our study found that the majority of the examined *nosZ* sequences are Clade I. If the two clades represent microbes with different N_2_O reduction kinetics, variations in community composition could influence the *in-situ* net N_2_O fluxes, causing ecological and environmental consequences (Hallin et al., 2018). Field studies are required to understand the biogeochemical impacts of the two clades on N_2_O consumption. In ETSP surface waters Clade II *nosZ* was found to be active and more abundant, implying a significant contribution to N_2_O consumption (Sun et al., 2017). However, in estuarine and coastal areas of China, Clade I *nosZ* was transcribed more actively than Clade II, despite being less abundant (Dai et al., 2022). Chesapeake Bay qPCR measurements show Clade II as ~2 orders of magnitude more abundance than Clade I, suggesting it dominates N_2_O consumption (Tang et al., 2022). Therefore, the two *nosZ* clades have varying biochemical impacts on N_2_O consumption, likely dependent on their environmental source.

In addition, analysis of co-occurrence of *nosZ* with other genes in the canonical denitrification pathway in cultivated microbes suggests that Clade I organisms were more often complete denitrifiers, with 83% of Clade I genomes also possessing *nirS* or *nirK* genes (which encode for ${\text{NO}}_{2}^{--}$ reduction), while about half of Clade II organisms lacked *nir* genes and are therefore “incomplete-denitrifiers” (Graf et al., 2014). This would suggest that a significant number of our *nosZ* sequences represent complete denitrifiers as the majority (~85% of sequences) are Clade I organisms. The denitrification pathway is often thought to be modular, but the extent of this modularity is unclear. If we assume that 83% of Clade I organisms and 50% of Clade II are complete denitrifiers (Graf et al., 2014), then ~76% of all sequences in the 5′ region and ~79% of in the 3′ region are complete denitrifiers. However, the estimate for the proportion of complete denitrifiers (Graf et al., 2014) used for this calculation was derived from cultivated organisms, therefore potentially introducing a bias. In addition, this may be an overestimate due to primer or sampling bias, especially since Clade II sequences were not recognized as distinct from Clade I prior to 2012. Nevertheless, the extent of the reduction of N_2_O to N_2_ by *nosZ* containing organisms may be clade dependent and should be investigated further.

#### 4.2.2 Taxonomic diversity

The 3′ region of the *nosZ* gene, which contained the majority of the total sequences, had a nearly equal number of archetypes representing Clade I and II (Figure 6); however, 86.5% of the sequences were in Clade I. This discrepancy suggests that there is a much greater level of diversity amongst Clade II organisms compared to Clade I. This difference might be in part due to a historical sampling bias favoring the discovery of Clade I sequences as Clade II was discovered over a decade later (Jones et al., 2012; Sanford et al., 2012). Regardless, a higher diversity for Clade II has been seen in other studies (Jones et al., 2014; Tsiknia et al., 2015; Wittorf et al., 2016) and is predicted due to a larger taxonomic breadth of Clade II sequenced genomes (Graf et al., 2014). In this study, the Clade II sequences from both the 5′ and 3′ regions of the *nosZ* gene come from a wider taxonomic range than observed for Clade I (Figure 4). The Clade II *nosZ* sequences in this study were identified as belonging to 20 distinct phyla, while Clade I sequences only included five phyla. Although Bacteroidota was the largest phylum found in Clade II, most of the total representative Clade II sequences were distributed across the remaining phyla. This is in contrast to Clade I sequences where the majority of representative sequences fell into one branch (i.e., Pseudomonadota/Proteobacteria). The high diversity of Clade II *nosZ* has made it impossible to create universal primers for PCR amplification. Chee-Sanford et al. (2020) tried to overcome this challenge by designing multiple primer sets to target different subclades. They assigned ten subclades of Clade II *nosZ* genes and were able to develop primer sets specifically targeting seven of those subclades. Even though Clade I *nosZ* has lower diversity, some of the existing primer sets for Clade I may not be suitable for high throughput analyses (Ma et al., 2019). Recently a new primer set was developed to target a broader range of Clade I sequences found in soils (Zhang L. et al., 2021). Due to the clear phylogenetic separation between Clades (Figures 2, 6) a truly universal *nosZ* primer set covering both clades would be impossible to design. In addition, it is important to note that while most sequences could be taxonomically assigned to phyla, 24% and 38% of the 3′ and 5′ representative sequences could not be taxonomically identified. This suggests that even with expanded and improved primer sets, it is likely not all *nosZ* sequences will be covered. Even the search for new *nosZ* genes in metagenomic datasets is hindered by the shear diversity of the gene at the DNA level.

#### 4.2.3 Ecological distribution (environmental sources) and niche differentiation

Due to the expanding view of *nosZ* diversity discussed above, little is known about the ecological distribution of the microbes capable of N_2_O reduction. In this study we analyzed the environmental sources and environmental distribution of the *nosZ* gene. Starting with a database of over 11,000 sequences, we were able to cluster and identify sequences by phylogenetic inference and classify them based on the environment from which they were obtained (Figures 2, 3, 6). Even though our data compilation discovered many previously unknown *nosZ* sequences, it is still important to recognize that many of the sequences included in this study were obtained by PCR amplification and clone libraries. Therefore, the patterns in the environmental sources and distribution that emerge in the following discussion are subject to PCR bias. Sequences that cannot be detected by PCR or even by HMMR searches at the DNA level may not exhibit the same patterns. With that caveat in mind, it is interesting to explore the biogeographical and ecological patterns in our expanded *nosZ* database.

We examined sequences from diverse biomes but focused primarily on those in marine and other aquatic environments. Most archetypes contained sequences from only one environmental source (82% and 75% for 5′ and 3′ regions), indicating they are unique to their environments (i.e., display biogeography). This supports previous findings of niche separation found between and within environmental sources and *nosZ* clades (Graf et al., 2014; Castro-González et al., 2015; Fuchsman et al., 2017). In the current study, archetypes from Clade II had a wider distribution across environmental sources than Clade I (Figures 2, 3, 6). This implies Clade II *nosZ* is better adapted to a broader range of environments than Clade I, likely due to the greater diversity observed. For both 5′ and 3′ regions of the *nosZ* gene, Clade I was predominately retrieved from marine sediment. This is consistent with results from a study of coastal sediment in which Clade I *nosZ* was found at a 10-fold higher abundance than Clade II (Wittorf et al., 2016). Unlike Clade I, a single environmental source did not dominate the Clade II sequences, which may be tied to the higher diversity of Clade II.

The widespread distribution of Clade II organisms, together with the high diversity, highlights the reduction of N_2_O as an ecologically important and favorable trait, not necessarily associated with canonical denitrification, but which may control net N_2_O emissions (Hallin et al., 2018). Physiological analyses of a few taxa suggest that bacteria with Clade II *nosZ* have a higher affinity for N_2_O compared to Clade I denitrifiers (Yoon et al., 2016). This potential mechanism for niche differentiation between clades would have ecological and environmental consequences. Oceanic OMZs are apparently dominated by Clade II organisms, selecting for efficient N_2_O scavenging which, in turn, would affect N_2_O removal rates (Bertagnolli et al., 2020). Clade I and II sequences from the 5′ region were nearly equally represented in the marine OMZs (7.8% and 5.3% respectively of sequences represented by each clade, Figures 2, 3A. However, in the 3′ region, which represents the majority of the sequences, Clade II (21.4%) was more highly represented than Clade I (8.2%) in the marine OMZs (Figures 3B, 6).

The archetypes containing sequences sourced from marine OMZs were examined at more localized scale to determine if the *nosZ* gene displayed regional, as well as environmental, biogeography. Archetypes containing marine OMZ sequences from both the 5′ and 3′ regions of the *nosZ* gene were mostly restricted to a particular OMZ (76% and 59.2% contained sequences sourced from only one OMZ, respectively; Table 1), although, it is possible that the sequences exist in other OMZs and have yet to be found. Distinct phylotypes have been detected between different OMZs and within an OMZ (Jayakumar et al., 2009; Castro-González et al., 2015; Bertagnolli et al., 2020). This further highlights the great diversity of *nosZ* containing organisms phylogenetically and possibly functionally. Several factors such as nutrient availability, oxygen concentration, redox state, etc., could help control this observed environmental distribution and needs to be examined further. For instance, differing *nosZ* community compositions was observed in the ETSP OMZ and was attributed to variations in oxygen concentrations (Sun et al., 2017). A study on salt marsh sediments observed variations in *nosZ* community structures at differing nitrogen loads (Kearns et al., 2015). Similarly, reef sediments have been shown to have redox-driven stratification of *nosZ*-containing bacterial communities (Gao et al., 2011). Phylogenetically distinct organisms have their own ranges and tolerances to environmental conditions, often allowing them to fill a niche. Phylogenetic variations in *nosZ* could further contribute to the observed environmental distribution as these variations may impact factors such as affinity constants (Yoon et al., 2016), oxygen tolerance, copper requirements, etc. The spread of the diverse taxa across environments has implications for N_2_O consumption rates between OMZs and is an active area of study.

Niche differentiation was also observed between the two predominant phyla, Bacteroidata and Pseudomonadota (Figure 5). For both 5′ and 3′ regions on the *nosZ* gene Bacteroidata sequences were primarily obtained from the marine water column and Pseudomonadota from marine sediment. Previous studies have shown Alphaproteobacteria, the Pseudomonadota class with the largest number of archetypes and sequences in this study, to be dominant in coastal marine sediments (Hunter et al., 2006; Wittorf et al., 2016). Niche differentiation between clades and phyla is unsurprising; however, the mechanism is unclear and requires further investigation. Niche partitioning beyond N_2_O affinity has been found in relation to oxygen availability, pH, and C:N ratios in soil (Domeignoz-Horta et al., 2015; Wittorf et al., 2016); however, niche partitioning in relation to environmental factors is understudied in marine and other aquatic environments.

### 4.3 Summary and future directions

*NosZ* containing organisms are found inhabiting diverse environments and cover a wide range of genera. The prevalence of these organisms has the potential to regulate global N_2_O emissions as *nosZ* is the only known biological sink of N_2_O. Although *nosZ* was first characterized in 1997 (Zumft), much remains unknown about the distribution, taxonomy, and biogeochemical impacts in the environment. Great diversity of the *nosZ* gene was found at the nucleotide level (such that sequences could not be well aligned); however, *nosZ* was highly conserved at the protein level and functionally remains the same despite evolving in diverse environments. This apparent functional constraint on the enzyme implies further ecological constraints, such as oxygen sensitivity or copper requirements; however, this has yet to be proven and requires further investigation. In addition, the extensive nucleotide sequence diversity found introduces detection and analytical challenges. Universal PCR primers for *nosZ* are not possible and, in turn, it is likely that *nosZ* diversity and environmental abundance are greatly underestimated. The ecological significance of this underestimation should be further examined. Instead of PCR primers, applying HMM to metagenomic studies is likely a better approach to identifying and characterizing new *nosZ* sequences, as done in this study.

We found Clade II to have a wider taxonomic range and distribution across environmental sources compared to Clade I. Differences in community composition, environmental distribution, and/or kinetics between Clade I and II containing organisms are likely to impact environmental N_2_O fluxes causing various ecological and environmental consequences. However, the extent of this is beyond the scope of this study, and field studies are required to understand the biogeochemical impacts of the two clades. The spread of the diverse taxa across environments has implications for N_2_O consumption rates.

This study examined the environmental distribution of the *nosZ* gene with a focus on marine and other aquatic environments to explore the breadth of environmental sources across both clades. In both 5′ and 3′ regions the majority of archetypes contain sequences obtained from only one environmental source. This suggests *nosZ* sequences, regardless of clade, is typically unique to an environment. Environmental factors such as oxygen levels, redox, nutrient availability, temperature, pH, etc., are likely involved in creating this observed separation between environmental sources. Clade I was mostly retrieved from marine sediments while Clade II sequences were derived from a much more diverse range of environmental sources including terrestrial, animal, wastewater systems, marine water column, hydrothermal vent systems, freshwater, and estuary systems. We found Clade I sequences primarily consisted of sequences from the Pseudomonadota phylum (predominately alphaproteobacteria) and Clade II was dominated by Bacteroidota sequences. These Bacteroidota sequences were mainly obtained from the marine water column while the Pseudomonadota were predominately obtained from marine sediments. The mechanism for the environmental distribution of *nosZ*, such as N_2_O affinity, nutrient availability, oxygen concentration, redox state, etc., is not well understood and should be examined further as it could explain variations in N_2_O fluxes. *NosZ* has the potential to regulate N_2_O emissions that contribute to ozone depletion and the greenhouse effect; this study characterized the diversity and environmental distribution of this gene to help further understand its biogeochemical impacts.

## Data availability statement

The original contributions presented in the study are included in the article/Supplementary material, further inquiries can be directed to the corresponding author.

## Author contributions

NI: Conceptualization, Data curation, Formal analysis, Investigation, Methodology, Visualization, Writing – original draft, Writing – review & editing. AJ: Data curation, Writing – review & editing, Investigation, Methodology. BBW: Conceptualization, Funding acquisition, Resources, Supervision, Writing – review & editing, Project administration.
