# Supplementary material for: Aquatic nitrous oxide reductase gene (nosZ) phylogeny and environmental distribution
Source: Front Microbiol. 2024 May 21;15:1407573. doi: 10.3389/fmicb.2024.1407573 (PMC11148229; doi:10.3389/fmicb.2024.1407573)
Supplement: Supplementary file 1 [file Data_Sheet_1.zip › Table 1 - 2024-05-07T175937.075.DOCX]

**Supplementary Table 1: Additional *nosZ* sequences added to database.** Cruise information, assembly or amplification method, accession numbers and number of sequences are listed.

| Cruise Location & Year | Assembly or amplification method | Accession numbers | *#* of  *nosZ* sequences |
| --- | --- | --- | --- |
| Arabian Sea 2007  R/V Roger Revelle | Metaspades | JGI: [1240937](https://genome.jgi.doe.gov/portal/AMALJGIDNA9_FD/AMALJGIDNA9_FD.info.html), [1240939](https://genome.jgi.doe.gov/portal/AMALJGIDNA10_FD/AMALJGIDNA10_FD.info.html), [1240941](https://genome.jgi.doe.gov/portal/AMALJGIDNA11_FD/AMALJGIDNA11_FD.info.html), [1240943](https://genome.jgi.doe.gov/portal/AMALJGIDNA12_FD/AMALJGIDNA12_FD.info.html) | 146 |
| ETNP 2016  R/V Ronald H. Brown | Metaspades | JGI: [1240921](https://genome.jgi.doe.gov/portal/AMALJGIDNA1_FD/AMALJGIDNA1_FD.info.html), [1240923](https://genome.jgi.doe.gov/portal/AMALJGIDNA2_FD/AMALJGIDNA2_FD.info.html),  [1240925](https://genome.jgi.doe.gov/portal/AMALJGIDNA3_FD/AMALJGIDNA3_FD.info.html), [1240927](https://genome.jgi.doe.gov/portal/AMALJGIDNA4_FD/AMALJGIDNA4_FD.info.html), [1240929](https://genome.jgi.doe.gov/portal/AMALJGIDNA5_FD/AMALJGIDNA5_FD.info.html), [1240931](https://genome.jgi.doe.gov/portal/AMALJGIDNA6_FD/AMALJGIDNA6_FD.info.html), [1240935](https://genome.jgi.doe.gov/portal/AMALJGIDNA8_FD/AMALJGIDNA8_FD.info.html) | 52 |
| ETNP 2018  R/V Sally Ride | Metaspades | JGI:  [1259837](https://genome.jgi.doe.gov/portal/AMALJGIDNA13_FD/AMALJGIDNA13_FD.info.html), [1259839](https://genome.jgi.doe.gov/portal/AMALJGIDNA14_FD/AMALJGIDNA14_FD.info.html), [1259841](https://genome.jgi.doe.gov/portal/AMALJGIDNA15_FD/AMALJGIDNA15_FD.info.html), [1259843](https://genome.jgi.doe.gov/portal/AMALJGIDNA16_FD/AMALJGIDNA16_FD.info.html), [1259845](https://genome.jgi.doe.gov/portal/AMALJGIDNA17_FD/AMALJGIDNA17_FD.info.html), [1259847](https://genome.jgi.doe.gov/portal/AMALJGIDNA18_FD/AMALJGIDNA18_FD.info.html), [1259851](https://genome.jgi.doe.gov/portal/AMALJGIDNA20_FD/AMALJGIDNA20_FD.info.html) | 41 |
|  | Primer based amplification | NCBI: PRJNA1107209 | 268 |
| ETSP 2013  R/V Nathaniel B. Palmer | Metaspades (Sun et al., 2019) | MG-RAST: mgm4842562.3, mgm4842563.3, mgm4842564.3, mgm4842565.3 | 16 |
| Chesapeake Bay 2020  R/V High R. Sharp | Primer based amplification | NCBI: PRJNA1107209 | 107 |
