# Supplementary material for: Aquatic nitrous oxide reductase gene (nosZ) phylogeny and environmental distribution
Source: Front Microbiol. 2024 May 21;15:1407573. doi: 10.3389/fmicb.2024.1407573 (PMC11148229; doi:10.3389/fmicb.2024.1407573)
Supplement: Supplementary file 1 [file Data_Sheet_1.zip › Figure Legends.docx]

Supplementary Material

# Supplementary Figure Legends:

**Supplementary Figure 1: Map of sample stations** from which novel *nosZ* sequences were obtained. The 2007 Arabian Sea (AS) is in purple, 2016 Eastern Tropical North Pacific (ETNP) in blue, 2018 ETNP in green, 2013 Eastern Tropical South Pacific in yellow, and 2020 Chesapeake Bay (CB) in orange. Map was made using MATLAB R2019b (MathWorks Inc.,2019).

**Supplementary Figure 2: Depiction of division of *nosZ* gene into 5’ (orange) and 3’ (yellow) regions compared to reference sequence from *Pseudomonas fluorescens* (green**). The region of overlap between the full 5’ and 3’ regions is ~42 bp. 70-mer regions were chosen on both sides. The 5’ 70-mer region is located around ~792-862 bp, and the 3’ 70-mer region is ~1322-1392 bp.

**Supplementary Figure 3: Rank abundance curve of 2,282 *nosZ* OTUs (circles) from all environments.** Red dashed line separates OTUs containing more than 2 sequences from those containing 2 or less, (unique/individual sequences) highlighted in yellow.
