# Supplementary figures and images for: Aquatic nitrous oxide reductase gene (nosZ) phylogeny and environmental distribution
Source: Front Microbiol. 2024 May 21;15:1407573. doi: 10.3389/fmicb.2024.1407573 (PMC11148229; doi:10.3389/fmicb.2024.1407573)

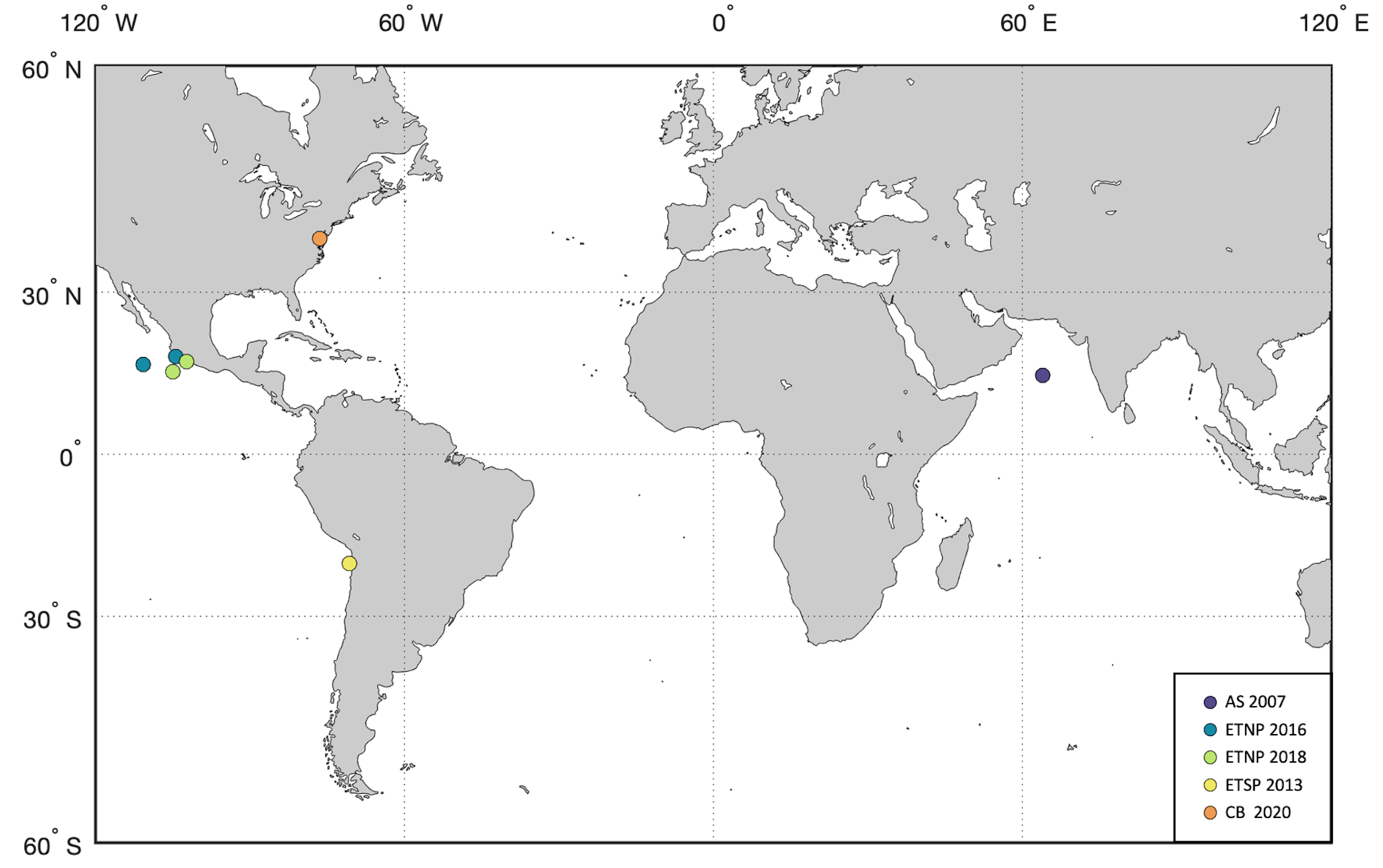

Supplement: Supplementary file 1 [file Data_Sheet_1.zip › Image 1 (12).PNG]

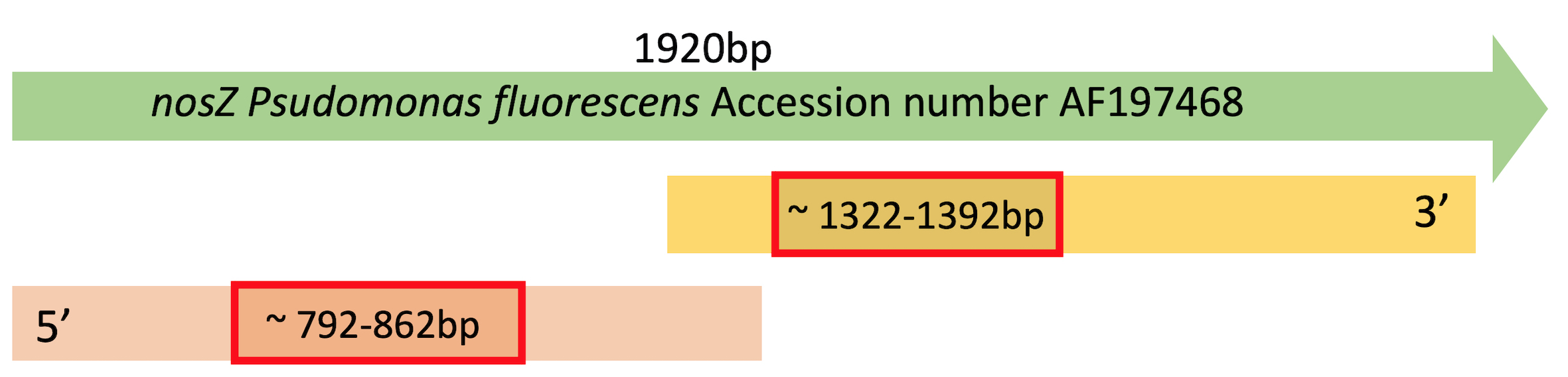

Supplement: Supplementary file 1 [file Data_Sheet_1.zip › Image 2 (53).JPEG]

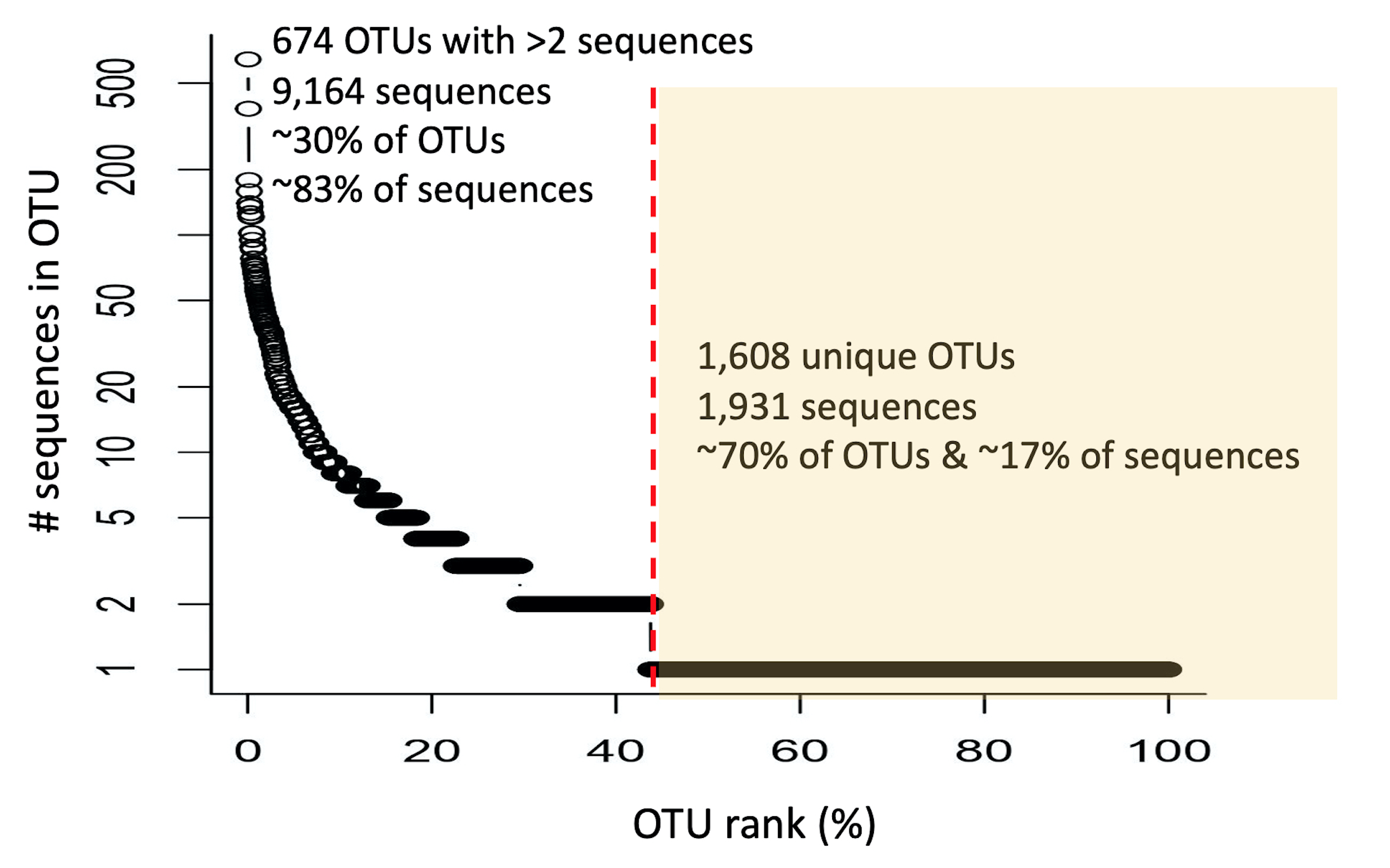

Supplement: Supplementary file 1 [file Data_Sheet_1.zip › Image 3 (25).JPEG]
